# Report of HKU-1 coronavirus nosocomial cluster in a city hospital in Japan during the coronavirus disease 2019 (COVID-19) pandemic

**DOI:** 10.1017/ash.2022.344

**Published:** 2023-01-17

**Authors:** Aoi Yogo, Shougen Sumiyoshi, Kazuaki Aoki, Hirofumi Motobayashi, Kentaro Tochitani, Shungo Yamamoto, Tsunehiro Shimizu

**Affiliations:** 1 Department of Infectious Disease, Kyoto City Hospital, Kyoto, Japan; 2 Department of Transformative Infection Control Development Studies, Osaka University Graduate School of Medicine, Suita, Osaka, Japan; 3 Division of Fostering Required Medical Human Resources, Center for Infectious Disease Education and Research (CiDER), Osaka University, Suita, Osaka, Japan

Human coronaviruses (HCoVs) cause respiratory tract infections. HCoV-229E, OC43, NL63, and HKU1 are common human coronavirus, and severe acute respiratory syndrome coronavirus (SARS-CoV), Middle East respiratory syndrome coronavirus (MERS-CoV), and SARS-CoV-2 are highly pathogenic.^1^ Common human coronaviruses are globally transmitted via inhalation of respiratory droplets with a 2- to 4-day incubation period, presumably accounting for 10%–15% of cases of common cold, especially in winter.^2^ HCoV-HKU1 causes mild, and self-limiting upper-respiratory diseases like common colds, bronchiolitis, and pneumonia, with symptoms such as rhinorrhea, fever, cough, and wheezing.^1^ More severe respiratory infections can occur in children or adults with underlying diseases and in the elderly.^3^

We identified the only report of a common human coronavirus outbreak with nosocomial transmission during the COVID-19 pandemic.^4^ Our HCoV-HKU1 outbreak occurred among healthcare workers (HCWs) in a single general ward at Kyoto City Hospital in Japan in January 2022, in a small, poorly ventilated break room where people were unmasked.

On January 19, 2022, the ward had 52 HCWs, and we identified 7 HCWs with respiratory symptoms, such as fever, rhinorrhea, and sore throats; no inpatients or other HCWs exhibited symptoms (Table 1). All 7 HCWs were confirmed to be SARS-CoV-2 negative via polymerase chain reaction (PCR) tests by nasopharyngeal swab samples (NPS; GeneXpert Xpress CoV-2 plus, Cepheid, Sunnyvale, CA). On January 20, 2022, we performed multiple respiratory panels using nasopharyngeal swab samples (BioFire FilmArray, bioMèrieux, Marcy-l'Étoile, France), a PCR-based multiplexed nucleic acid test for common respiratory microorganisms. Of the 7 HCWs, 6 tested positive for HCoV-HKU1.

All 7 HCWs had been assigned to the same general ward and had been wearing surgical masks while caring for patients. We identified a shared break room as a potential transmission route; 6 of the HCWs had used this room unmasked for a few days before their symptom onset. It was difficult to sufficiently distance from others in the small and poorly ventilated room. We could not identify the accurate transmission route among HCWs, and we did not investigate inpatients and other HCWs on the ward because they had no respiratory symptoms and were less likely to be transmission sources. All respiratory symptoms in the HCoV-HKU1–postive HCWs improved, and they returned to work within a week after their symptom onset. An investigation revealed that a nurse on the same ward had returned from vacation a few days prior to the symptom onset in the 7 HCWs and had worked with rhinorrhea. Because her symptoms had resolved at that point, we did not test her for HCoV-HKU1, but we speculated that she was the index case.

## Discussion

HKU1 coronaviruses cause common colds in healthcare populations, but they can have more critical impacts on children, elderly persons, and those with underlying diseases.^5^ A case series on community-acquired pneumonia at multiple hospitals in Hong Kong showed that 8 of 10 patients confirmed with HCoV-HKU1 had underlying diseases, and 2 of them died.^6^ Our HCWs had no underlying diseases, and no patients on this ward were symptomatic. However, common human coronavirus spreading at a hospital could be critical for elderly patients with underlying diseases. More careful nosocomial infection control is required, even for milder common respiratory viruses.

Currently, we are still advised to wear masks, to keep our distance from others (2 m or 6 feet) to avoid close contact, to avoid poorly ventilated spaces and crowds, and to wash our hands often. Universal masking is required for all patients, visitors, and personals in healthcare settings to reduce transmission of SARS-CoV-2 from unsuspected virus carriers.^7^ Furthermore, the US Center for Disease Control and Prevention (CDC) recommends wearing a well-fitting mask indoors in public spaces, regardless of vaccination status or individual risk, because surgical masks can prevent transmission of human coronavirus—as well as influenza—from individuals with acute respiratory symptoms.^8,9^ Our assessment of the reported hospital outbreak shows that transmission occurred when the symptomatic HCWs were unmasked. The BNT162b2 vaccine has been reported to induce antibodies against the spike protein of human seasonal β coronavirus (HKU1 and OC43), and all 7 HCWs, including the 6 confirmed positive by PCR, had received the BNT162b2 vaccine at least twice. However, this vaccination did not prevent their HKU1 infections.^10^ Universal masking is therefore extremely important for infection control at medical institutions as new SARS-CoV-2 variants that decrease the efficacy of the COVID-19 vaccine continue to emerge.

Our study had several limitations. We drew conclusions about the index case in a clinical situation without access to laboratory evidence. Therefore, we could not clearly determine the incubation time since exposure to the index case and onset of symptoms in our 7 secondarily infected HCWs. This investigation was made more difficult by the fact that HCW activities outside the hospital ward were not known to us. Ideally, epidemiologic information should be collected in real time and supplemented by laboratory findings.

In conclusion,

we report a nosocomial HCoV-HKU1 outbreak among HCWs on a general ward. The first important implication of this outbreak is the possibility of common respiratory viruses circulating at hospitals, which might pose a severe risk for the elderly or those with underlying respiratory diseases. Another point our report emphasizes is the higher transmission risk when unmasked, which could be a pitfall during mealtimes and breaks. We should screen HCWs with respiratory symptoms.

**Fig. 1.**
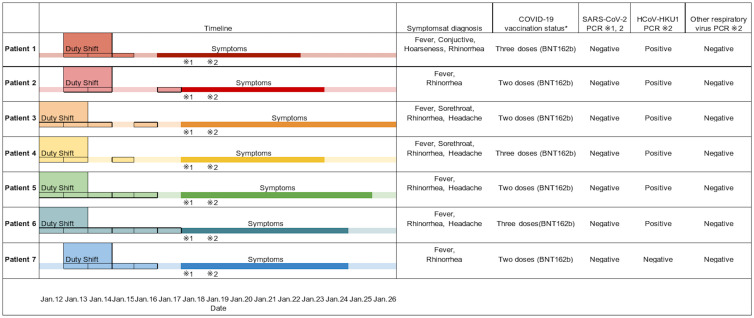
COVID-19, coronavirus disease 19; SARS-CoV-2, severe acute respiratory syndrome coronavirus 2; HCoV-HKU1, human coronavirus-HKU1. *A person vaccinated with 2-dose vaccine had completed the primary series of COVID-19 vaccine >14 days before illness onset. A person with a 3-dose vaccine had completed the booster vaccine after primary series of COVID-19 vaccine. Note. Respiratory multiple PCR tests included adenovirus, SARS-CoV2, human coronavirus (229E, HKU1, NL63, OC43), human metapneumovirus, influenza virus A/B, parainfluenza virus, respiratory syncytial virus, human rhinovirus (type 1A)/enterovirus (D68), *Bordetella* parapertussis, *Bordetella* pertussis, *Chlamydophila pneumonia*, and *Mycoplasma* pneumonia.
